# Servant Leadership and Creativity: A Study of the Sequential Mediating Roles of Psychological Safety and Employee Well-Being

**DOI:** 10.3389/fpsyg.2021.807070

**Published:** 2022-02-04

**Authors:** Wenxian Wang, Seung-Wan Kang, Suk Bong Choi

**Affiliations:** ^1^College of Business, Gachon University, Seongnam, South Korea; ^2^College of Global Business, Korea University, Sejong City, South Korea

**Keywords:** servant leadership, psychological safety, employee well-being, creativity, conservation of resources theory

## Abstract

With today’s increasingly dynamic and competitive business environment, creativity is critical for enterprises to enhance their competitiveness. Companies today invest and seek new ways to enhance creativity of employees within the organization. Our study describes the effects of servant leadership, psychological safety, and employee well-being on creativity under the conservation of resources theory. We used a sample of 252 full-time employees in the United Kingdom who had been recruited online and collected their data for analysis. We conducted confirmatory factor analyses to test the validity of the measurement model and regression to evaluate the direct effects. Subsequently, we used bootstrapping to confirm mediation and serial mediation effects. The results showed that servant leadership was positively related to creativity and that psychological safety and employee well-being were serial mediators between them.

## Introduction

Today, with the broadening of globalization and information technology and the rapid technological developments, there is competition for organizations’ focus among information, knowledge, and creativity. Today’s organizations are operating in an unstable business environment that requires greater effort to achieve organizational innovation (Tierney and Farmer, 2011). Creativity is a key source of competitive advantage and sustained success in the current dynamic and highly competitive business environment (Hughes et al., 2018). Most of the previous studies focused on organizational innovation, and research on employee creativity has remained in its early stages until recently; in the new century, employee creativity began to receive increasing attention (Tierney and Farmer, 2011). Employees who are creative at work generate ideas that benefit the organization (Shalley and Gilson, 2004). As the main body of enterprise innovation, employee creativity can effectively promote enterprise development, and because creativity promotes innovation, growth, and competitiveness (Gong et al., 2013), most organizations invest heavily in finding effective ways to encourage employee creativity (Liu et al., 2012).

However, in practice, not all employees are willing to do this unconditionally: Creativity is risky and requires challenging the *status quo*, which heightens unpredictability (George, 2007). New ideas can fail, leading to self-blame and even rejection by others. Based on these findings that creativity is challenging and consumes employees’ personal resources, we applied the conservation of resources theory (COR; Hobfoll, 1989) in this study to explain the antecedents of employee creativity.

According to COR theory (Hobfoll et al., 2018), lack of resources can lead to pressure and employee burnout, and employees with few resources to spare avoid investing their limited resources in activities perceived as non-productive, such as creativity. Employees with more resources, for instance, low emotional exhaustion, are more inclined to take on the challenge of creativity. Therefore, we investigated employee well-being as an influence on creativity.

Psychological safety is a resource (Singh et al., 2018) that creates comfort. In the workplace, employees who feel psychological safety will feel freer to express themselves and perceive less risk in doing so; environments of psychological safety reduce employee stress and burnout and increase employee well-being. In contrast, employees who do not perceive psychological safety in their environments fear taking risks and possibly making mistakes that may be generated by creativity, and their higher stress has negative impacts on their well-being. Based on COR theory, we consider psychological safety an influence on creativity and employee well-being.

Leaders can play a key role in employees’ psychological safety (Dirik and Intepeler, 2017). Edmondson (2004) found that leaders’ openness, accessibility, and availability can form a sense of psychological safety among their followers. Servant leadership puts followers first and focuses on their development (Hoch et al., 2018). In the workplace, servant leadership manifests as providing employees with job resources, talent activation, and career opportunities to develop their skills, activate their talent and empower them (van Dierendonck, 2011), and such efforts promote employees’ psychological safety. Therefore, we consider servant leadership an influence on psychological safety.

Although previous researchers have found correlations between servant leadership and creativity (Neubert et al., 2008, 2016; Yoshida et al., 2014; Linuesa-Langreo et al., 2016; Williams et al., 2017; Yang et al., 2019; Ruiz-Palomino and Zoghbi-Manrique-de-Lara, 2020), others have concluded that the relationship is uncertain (Newman et al., 2018). Therefore, it is necessary to further explore the mechanism of whether and how servant leadership promotes creativity (Liden et al., 2014).

Eva et al. (2019) proposed a nomological network that considered psychological safety a mediating variable and well-being an outcome variable. Based on this research and the above findings, we attempted here to use COR theory to explain the psychological mechanism of how servant leadership positively affects employees’ psychological safety, thereby improving employee well-being, and thus promoting employee creativity.

In summary, it is not easy for organizations to improve the creativity of their employees. We attempted to search for antecedents that can improve creativity according to previous studies. Based on COR theory, we found that employee well-being, psychological safety, and servant leadership affected creativity, and therefore, we expected to find the pathways and mechanisms of how these variables affect creativity in the workplace through this study.

To investigate these questions, we recruited participants for the survey and conducted two survey waves at an interval of 6 weeks between them; we then collected and analyzed the data. In the following section, we show how we used COR theory to hypothesize the psychological mechanism between servant leadership and creativity. We respectively present research hypotheses, methodology, and empirical results of this study. Finally, we discuss the theoretical and practical implications of this paper’s findings and propose directions for future research.

## Theoretical Background and Hypotheses

### Servant Leadership and Psychological Safety

Greenleaf (1977) first proposed the concept of servant leadership. The same author later elaborated in *The Servant as Leader* (Greenleaf, 2007) that servant leadership is service-oriented to meet the needs of subordinates. Researchers have proposed a number of definitions of servant leadership, including (Greenleaf, 1977), who described a leader who perseveres to serve first rather than lead. Later, van Dierendonck (2011) expanded to say that servant leaders meet subordinates’ needs, provide them with learning opportunities, and improve their self-management capacities. Extant research defined servant leadership as an other-oriented approach that entailed prioritizing followers’ needs and leaders reorienting their concern for themselves toward concern for others in the organization (Eva et al., 2019; Elche et al., 2020).

Kahn (1990) defined psychological safety as a condition whereby individuals believe they can express their true selves freely without fear of harming their career, status, or self-image, while Brown and Leigh (1996) defined it as the degree to which an individual psychologically perceives safety in the organization’s environment. Edmondson (1999, 2004) defined the concept as a common belief of employees that organizational members trust and respect each other, the workspace is safe for interpersonal risk-taking, and they will not be punished for sharing opinions, taking risks, or making mistakes.

Based on COR theory, individuals with more resources have a stronger ability to possess and conserve other resources, whereas those with fewer resources have a weaker ability to possess and conserve resources (Hobfoll et al., 2018). In the workplace, when leaders show care and concern for employees, help them grow, and give them support, they provide resources to employees and contribute to generating employees’ psychological safety (Iqbal et al., 2020).

Servant leadership helps employees to acquire resources (Zoghbi-Manrique-de-Lara and Ruiz-Palomino, 2019; Ruiz-Palomino et al., 2021). Servant leadership is oriented toward helping and guiding employees, showing compassion, healing, awareness, persuasion, management, and commitment to subordinates’ growth (Russell, 2001). Servant leadership encourages employees to ask questions and take on challenges and rewards them with promotions for doing so (Karatepe et al., 2019). These components of paying attention to employees’ needs and fostering their self-development allow employees the space to take risks and make mistakes and also provide them with the resources to feel this freedom. Indeed, Erkutlu and Chafra (2019); Chughtai (2016), and Schaubroeck et al. (2011) demonstrated positive correlations between servant leadership and psychological safety. Given the above findings, we proposed the following hypothesis:

**Hypothesis 1 (H1).** Servant leadership is positively related to psychological safety.

### The Mediating Role of Psychological Safety in the Relationship Between Servant Leadership and Employee Well-Being

Warr (1987) defined employee well-being as employees’ overall evaluations of the quality of their work experiences and functions, and Ryan and Deci (2001) defined well-being as reflecting optimal mental function and experience. Grant et al. (2010) defined employee well-being as happiness gained from work including core influences and satisfaction with intrinsic and extrinsic work values, and Bakker and Oerlemans (2012) defined well-being in general as personal satisfaction with life experiences including positive and negative emotions.

According to COR theory (Halbesleben et al., 2014), when individuals have fewer resources at work, they are more prone to stress and burnout, resulting in low employee well-being, whereas when individuals have more resources at work, their stress and burnout decrease and their well-being increases. Moreover, employees who perceive high psychological safety in the workplace perceive support and respect (Chen et al., 2014) and feel more freedom to express themselves (Edmondson and Lei, 2014), while employees who feel less psychological safety spend time and energy confronting interpersonal risks (Edmondson, 2018), and their stress results in consumption (Yam et al., 2016), which further reduces resources, increases stress and decreases well-being. Numerous other researchers have demonstrated strong support, including empirical evidence, for correlations between psychological safety and employee well-being (Sharifirad, 2013; Hasan and Kashif, 2020; Xu et al., 2020; Zhang and Song, 2020). Others highlighted that environments of psychological safety promote positive employee attitudes and emotions (Kirk-Brown and Van Dijk, 2016). In short, and in keeping with COR theory, psychological safety can be considered a resource (Singh et al., 2018) that contributes to employee well-being.

Meanwhile, as discussed above, servant leadership entails providing employees with work resources, activating their talents, and providing career development opportunities (van Dierendonck, 2011), and leaders who prioritize their employees’ needs and support them in their work promote the psychological safety that contributes to improving employee well-being. Indeed, previous researchers identified positive correlations between servant leadership and employee job satisfaction (Mayer et al., 2008; Schneider and George, 2011). In particular, Chen et al. (2013) and Gotsis and Grimani (2016) found positive correlations between servant leadership and employee happiness because servant leaders served their followers and prioritized their well-being over achieving short-term organizational goals. Servant leaders also improve employee well-being just by creating positive working atmospheres (Neubert et al., 2008; Jaramillo et al., 2009; Black, 2010). Given these relationships, we speculate that psychological safety is the mediating factor between servant leadership and employee well-being.

Schepers et al. (2008) demonstrate that social support and psychological safety promote well-being. Frazier et al. (2017) and Newman et al. (2017) found that psychological safety played a mediating role in leadership behavior and team performance, and Lyu (2016) showed that psychological safety played an intermediary role between organizational justice and work engagement. Given these relationships, we propose the following hypothesis:

**Hypothesis 2 (H2).** Psychological safety mediates the relationship between servant leadership and employee well-being such that servant leadership enhances psychological safety and psychological safety leads to greater employee well-being.

### Servant Leadership and Creativity

Amabile (1983) defined creativity as the ability to create novel and useful ideas, and Oldham and Cummings (1996) defined it as novel and useful products, ideas, or procedures that can help organizations develop and succeed. Shalley et al. (2004) also defined creativity as developing novel and potentially useful ideas, and George (2007) defined it as employees proposing new ideas to improve workflow and enhance efficiency.

In accordance with COR theory (Hobfoll et al., 2018), individuals have a tendency to accumulate, preserve, nurture, and protect valued resources because resources lost are far more important than resources gained. Creativity can be challenging and can destroy the original work balance (George, 2007), resulting in the risk of losing work resources. Therefore, employees will try to prevent the loss of resources by avoiding creativity. Service leadership prioritizes the needs of subordinates over the needs of managers (Greenleaf, 2007) and provides them with resources (van Dierendonck, 2011; Liden et al., 2014). Based on these findings, we used COR theory to investigate how servant leadership influences employee creativity.

Meanwhile, servant leadership encourages the interests of subordinates rather than focuses on the interests of competitors (Hoch et al., 2018). It entails helping employees succeed and grow, providing them with sufficient resources that they will be open to in creativity and challenge without fear of resource loss. In previous studies, servant leadership was related to group creativity (Linuesa-Langreo et al., 2016) and promoted creativity through servant attitude (Ruiz-Palomino and Zoghbi-Manrique-de-Lara, 2020). Servant leadership also encourages workplace spirituality, which can enhance employee creativity (Williams et al., 2017). Servant leadership enhances employee creativity with the mediator of creative self-efficacy (Yang et al., 2017). Following these findings, we hypothesized the following:

**Hypothesis 3 (H3).** Servant leadership is positively related to creativity.

### Psychological Safety and Creativity

According to COR theory, it is critical for individuals to replenish depleted resources, particularly for people who already have few resources or who have lost resources, the new resources provided are more important for them to replenish resources or counteract resource losses (Hobfoll et al., 2018). Therefore, when resources are replenished, individuals are more willing to engage in activities that could cause resource loss.

Workplace creativity is risky in that it requires challenging the *status quo* and unsettling things (George, 2007), which can increase unpredictability and cause resource loss, and, employees need psychological resources to cope with risks and challenges (Spreitzer et al., 2012; Carmeli et al., 2014). Because psychological safety is a resource (Singh et al., 2018), it will help employees compensate for the resources lost to challenging creativity, increasing their willingness to be creative.

In previous research, psychological safety was connected with employee creativity through organizational identification (Liu et al., 2016) and with follower creativity by the moderator of knowledge sharing (Wang et al., 2018). Team psychological safety has a positive impact on team creativity (Lee et al., 2018), and psychological safety is related to employee creativity mediated by work engagement (Liu and Ge, 2020). The psychological safety promoted by inclusive leadership could enhance subordinates’ creativity (Zhu et al., 2020). These findings led to the following hypothesis:

**Hypothesis 4 (H4).** Psychological safety is positively related to creativity.

### Sequential Mediating Role of Psychological Safety and Employee Well-Being Between Servant Leadership and Creativity

According to COR theory (Halbesleben et al., 2014), people use the key resources they have to cope with stressful situations in the work environment on the one hand, and actively build and protect their existing resource pool to cope with possible future stressful situations on the other. Employees may invest in resources with the expectation of acquiring new resources in the future to make up for possible future resource losses.

Employees who have more psychological resources cope better with pressure and are more willing to accept challenges and risks, and they also contribute more ideas and perform effectively at work. Meanwhile, effective servant leadership provides employees with both tangible and intangible resources, gives them autonomy and decision-making power, emphasizes their benefits, and promote their growth and success (van Dierendonck, 2011; Liden et al., 2014), but previous researchers have also connected servant leadership in organizations with employees’ creativity (Neubert et al., 2008). Yoshida et al. (2014) contended that servant leadership stimulates employee creativity via relational identification, and Liden et al. (2014) maintained that because servant leadership empowers followers and focuses their growth and development, it can promote their creativity.

Extant research has established that individuals with positive emotions are more likely to help others than are people with negative or neutral emotions (Carlson et al., 1988), in the workplace, employees who feel positive emotions often exhibit extra-role behaviors (George, 1991). Sonnentag (2015) also found that employee well-being encouraged taking on extra-role behaviors such as creativity to support the organization to achieve common goals, and Miao and Cao (2019) found that work well-being positively affected employees’ creativity.

**FIGURE 1:**
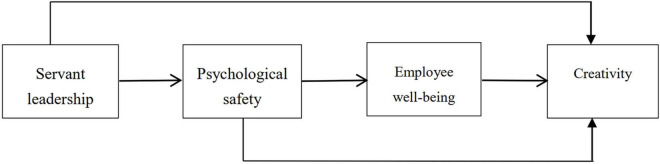
Hypothesized research model.

Khan et al. (2020) used a serial mediation model to find that servant leadership led to trust and then to job crafting, which promoted employees’ innovative work behaviors. Based on these above findings, we proposed a serial mediation research model in Figure 1 and the following hypothesis:

**Hypothesis 5 (H5).** Psychological safety and employee well-being serially mediate the relationship between servant leadership and creativity such that servant leadership enhances psychological safety, which in turn increases employee well-being, and the increased well-being enhances employee creativity.

## Materials and Methods

### Sample

For this study, we recruited participants online by using the online panel platform which is United Kingdom-based and specifically designed for academic research (Palan and Schitter, 2018) that produces high quality, reliable sample data (Peer et al., 2017). And online panel data have been shown at least the same quality as traditional field samples (Walter et al., 2019) and previous researchers have used this process (Neubert et al., 2008; Mai et al., 2021). We used multi-time data collection to reduce common method bias and embedded attention checks (Marjanovic et al., 2014; Peer et al., 2014; Cheung et al., 2017) to screen for non-conscientious respondents. Attendance checks are an easy way to see if the participants are following your study instructions (Oppenheimer et al., 2009), such as through giving a study’s participants explicit instructions for completing a particular task (e.g., “click ‘Strongly disagree’ to answer this question.”). Following previous research on employee well-being, we set a 6-week interval between the two waves of this survey (Tong et al., 2019).

In the first wave, the employees rated their perceptions of their supervisors’ servant leadership and psychological safety and answered control questions related to gender, age, education, organization tenure, and interaction frequency. We received 321 surveys, and after we deleted missing data and failed attention checks, 299 remained. In the second wave, employees rated their well-being and creativity. We sent the wave 2 questionnaires to the 299 participants of the last wave and received 254; 252 surveys remained after we deleted the failed attention checks, for a response rate of 84.3%.

All participants in the sample were from the United Kingdom where the online panel platform is located, and they are English native speakers. Their average age was 38.61 years (SD = 9.80), and 58.33% of respondents were women; 41.67% were male. By education background, 14.68% had completed high school, 22.22% had college diplomas, 42.86% held bachelor’s degrees, 15.87% had master’s degrees, and 4.37% held doctorates. Respondents had been with their organizations for a mean of 8.23 years (SD = 7.43) and reported interacting with their supervisors an average of 22.16 times (SD = 24.19) in a given week. Table 1 presents the details.

**TABLE 1:** Demographic variables.

Demographics	Items	No.	Percentage
Gender	Male	105	41.67
	Female	147	58.33
Age	21–30	56	22.22
	31–40	103	40.87
	41–50	61	24.21
	51–60	25	9.92
	61–	7	2.78
Final education level	High school	37	14.68
	College Diploma(without bachelor degree)	56	22.22
	Bachelor	108	42.86
	Master	40	15.87
	Doctor/Ph.D.	11	4.37
Organization tenure	0–1	18	7.14
	1–3	50	19.84
	3–5	41	16.27
	5–7	36	14.29
	7–10	31	12.30
	10–15	40	15.87
	15–20	17	6.75
	20–30	12	4.76
	30–	7	2.78

### Measures

#### Servant Leadership

We used Liden et al.’s (2015) seven-item scale to measure servant leadership. Respondents rated items on five-point Likert scales that ranged from 1 (*strongly disagree*) to 5 (*strongly agree*). A sample item is “My supervisor gives me the freedom to handle difficult situations in the way that I feel is best.” Cronbach’s alpha was 0.84 (see Appendix A for all items).

#### Psychological Safety

We used May et al. (2004) three-item scale to measure psychological safety. Responses were rated on Likert scales from 1 (*strongly disagree*) to 5 (*strongly agree*). A sample item is “I’m not afraid to express my opinions at work.” Cronbach’s alpha was 0.74 (see Appendix A for all items).

#### Employee Well-Being

We used Brunetto et al.’s (2011) four-item scale to measure employee well-being, and the items were rated on five-point Likert scales ranging from 1 (*strongly disagree*) to 5 (*strongly agree*). A sample item is “Overall, I think being a current job worker fulfills an important purpose in my work life.” Cronbach’s alpha was 0.83 (see Appendix A for all items).

#### Creativity

Creativity generally only refers to generating new ideas, which employees can freely control (Axtell et al., 2000). Previous research indicates that creativity measurement might be best suited to self-report by employees because peers and supervisors might not notice employees’ creative contributions except for those who make active efforts to gain recognition (Janssen, 2000), although other researchers have found marked convergence between individuals’ self-reported creativity and their peers’ and supervisors’ ratings of their creativity (Amabile et al., 2005; Shalley et al., 2009; Moneta et al., 2010). Thus, for our study, we measured creativity using Neubert et al.’s (2016) three-item self-report scale. These items were also rated on five-point Likert scales ranging from 1 (*strongly disagree*) to 5 (*strongly agree*). A sample item is “I generate creative ideas at work.” Cronbach’s alpha was 0.88 (see Appendix A for all items).

#### Control Variables

We controlled for the variables of age, education, and organization tenure to account for demographic differences in predicting employee well-being and creativity (Yoshida et al., 2014; Williams et al., 2017; Sprigg et al., 2018), measuring age and organization tenure in years as continuous variables and gender as a dichotomous variable (0 = female, 1 = male). Education attainment had six categories mentioned above: elementary school, high school, college diploma, bachelor, master, and PhD.

Because Howell et al. (2005) found that interaction frequency also influences employee performance, we included interaction frequency as a control variable measured with the response to how many times they interacted with their supervisors in a week including in person, by phone, by email, or otherwise.

### Common Method Bias

To reduce common method bias, we employed multi-time data collection, but because the same respondents measured all the variables, bias could still have been generated that could have resulted in false internal consistency and created potentially misleading results (Chang et al., 2010). Following Podsakoff et al. (2003), we conducted Harman’s single-factor test by loading all the items for the research constructs into an exploratory factor analysis. The results showed that no single factor explained more than 40% of the variance (Fuller et al., 2016), which indicated that common method bias had not significantly influenced the validity of the results.

### Analytical Strategy

We performed all statistical analyses using STATA 15.1 (Stata Corp., College Station, TX, United States). First, we conducted confirmatory factor analysis to calculate the validity of the study variables and ran a chi-squared model comparison; all variables were captured and analyzed at the individual level. Then, we used regression analyses to test the direct effects and bootstrapping analysis with 10,000 resamples to confirm the indirect effect (Preacher and Hayes, 2008).

## Results

### Descriptive Statistics

Table 2 presents the variable means, standard deviations, and correlations. Servant leadership correlated significantly with psychological safety (*r* = 0.39, *p* < 0.001), employee well-being (*r* = 0.42, *p* < 0.001), and creativity (*r* = 0.27, *p* < 0.001). Psychological safety also correlated significantly with employee well-being (*r* = 0.33, *p* < 0.001) and creativity (*r* = 0.18, *p* < 0.01). In the regression analyses, the variance inflation factors of all independent variables were below 10, indicating the absence of any multicollinearity problem (Aiken and West, 1991).

**TABLE 2:** Cronbach’s alphas, means, standard deviations, correlations, and reliabilities.

Variable	Alpha	Mean	SD	1	2	3	4	5	6	7	8	9
1.Gender(0 = F,1 = M)^a^		0.42	0.49	–								
2.Age		38.61	9.80	0.06	–							
3.Education		3.73	1.04	–0.01	–0.04	–						
4.Organization tenure^b^		8.23	7.43	0.08	0.49***	–0.17**	–					
5.Interaction frequency^c^		22.16	24.19	0.05	–0.02	–0.21***	0.01	–				
6.Servant Leadership	0.84	3.18	0.80	–0.04	–0.12	0.09	–0.11	0.20**	–			
7.Psychological Safety	0.74	3.93	0.86	–0.03	–0.04	–0.07	–0.06	0.13*	0.39***	–		
8.Employee Well-being	0.83	3.40	0.88	–0.02	0.07	0.13*	–0.06	0.04	0.42***	0.33***	–	
9.Creativity	0.88	3.40	0.94	0.03	–0.03	0.12	0.03	0.05	0.27***	0.18**	0.30***	–

n = 252. *p < 0.05. ^a^F = Female; M = male. ^b^Tenure = number of years. ^c^Frequency = Times in a week. **p < 0.01; ***p < 0.001 (two-tailed test).

### Confirmatory Factor Analysis and Chi-Square Difference Test

According to Marsh et al. (2004), a model has good fit to the data when the comparative fit index (CFI) and Tucker-Lewis index (TLI) are 0.90 or above, and the root-mean square error of approximation (RMSEA) is less than or equal to 0.08. For this study, the four-factor hypothesis model showed the best fit indices (χ^2^ = 269.81; df = 113; CFI = 0.92; TLI = 0.90; RMSEA = 0.07). Table 3 shows the results from comparing the other models.

**TABLE 3:** Measurement model fit statistics.

Measurement model	χ^2^	Df	CFI	TLI	RMSEA	Δχ^2^	Δdf
Baseline (hypothesized) four-factor model	269.81	113	0.92	0.90	0.07		
Alternative 1 (three-factor model)^a^	646.00	116	0.73	0.68	0.14	376.19***	3
Alternative 2 (two-factor model)^b^	829.69	118	0.64	0.58	0.16	559.88***	5
Alternative 3 (one-factor model)^c^	1,104.17	119	0.50	0.42	0.18	834.36***	6

n = 252. ^a^A three-factor model with employee well-being and creativity on the same factor. ^b^A two-factor model with employee well-being, creativity, and psychological safety. ^c^A one-factor model with servant leadership, employee well-being, creativity and psychological safety. ***p < 0.001 (two-tailed test).

### Hypothesis Testing

H1 predicted that servant leadership positively relates to psychological safety, and Table 4 indicates a positive and significant relationship (β = 0.42, *p* < 0.001; Model 1) after we controlled for gender, age, education, organization tenure, and interaction frequency. We conclude that H1 was supported.

**TABLE 4:** Regression analysis results and bootstrapped indirect effects.

Main effects	Psychological safety	Employee well-being		Creativity		
	Model 1	Model 2	Model 3	Model 4	Model 5	Model 6
Gender	–0.02	–0.01	–0.00	0.06	0.07	0.07
Age	0.00	0.01*	0.01*	–0.01	–0.00	–0.01
Education	–0.09	0.07	0.09	0.14*	0.10	0.09
Organization tenure	–0.00	–0.01	–0.01	0.01	0.01	0.02
Interaction frequency	0.00	–0.00	–0.00	0.00	0.00	0.00
Servant Leadership	0.42***	0.47***	0.38***		0.31***	0.17*
Psychological Safety			0.21**	0.20**		0.07
Employee well-being						0.24**
F	8.27***	10.53***	10.96***	2.68*	4.10***	4.86***
R2	0.17	0.21	0.24	0.06	0.09	0.14
ΔR2			0.03		0.03	0.05

**Indirect effects**	**Estimate**		**Lower limit**		**Upper limit**	

SL → PSF → EWB	0.09		0.03		0.16	
PSF → EWB → CR	0.10		0.04		0.17	
SL → PSF → EWB → CR	0.02		0.01		0.05	

n = 252. *p < 0.05; **p < 0.01; ***p < 0.001 (two-tailed test) for the standardized regression coefficients.

H2 posited that psychological safety would mediate the relationship between servant leadership and employee well-being such that servant leadership would enhance psychological safety and psychological safety would lead to better employee well-being. In bootstrapping to test the mediating effect of servant leadership on employee well-being through psychological safety, the observed coefficient effect was 0.09, and the 95% bias-corrected bootstrap confidence interval (CI) did not include 0 [0.03,0.16]. Thus, we consider H2 supported.

H3 proposed that servant leadership is positively related to creativity. After controlling gender, age, education, organization tenure, and interaction frequency. The results of Table 4 show there is a positive relationship between servant leadership and creativity (β = 0.31, *p* < 0.001; Model 5). So, the H3 was supported.

And H4 posited that psychological safety has a positive relationship with creativity. After we controlled gender, age, education, organization tenure, and interaction frequency, Table 4 indicates psychological safety is positively related to creativity (β = 0.20, *p* < 0.01; Model 4). Hence, the H4 was supported.

H5 predicted that psychological safety and employee well-being would serially mediate the relationship between servant leadership and creativity such that servant leadership would enhance psychological safety, which would in turn increase employee well-being, and the increased employee well-being would enhance creativity. Indirect testing with bootstrapping produced a coefficient of 0.02, and the 95% bias-corrected bootstrap CI also excluded zero [0.01, 0.05]. Results indicate that H5 was supported.

## Discussion

Employee creativity is crucial to organizations’ competitiveness (Hughes et al., 2018). In the workplace, leaders have impacts on employees that affect their creativity. Based on COR theory, we assumed certain effects of the servant leadership style on employee creativity factoring in direct effect and serial mediating effects of psychological safety and employee well-being. Our findings support our hypotheses, and we discuss the theoretical and practical implications of the findings below.

### Summary

The results of this study contribute to the existing literature by our having examined how servant leadership impacts employee performance. Using COR theory, our research shows that servant leadership promotes psychological safety and has a positive impact on employee well-being, psychological safety mediates the relationship between servant leadership and employee well-being, servant leadership and psychological safety both correlate positively with creativity, and servant leadership has a positive impact on employee creativity through two serial mediators: psychological safety and employee well-being. This study makes a positive contribution to understanding the psychological mechanism of how servant leadership promotes employees’ creativity.

### Theoretical Implications

First, with this study, we responded to previous scholars’ (Neubert et al., 2008; Liden et al., 2014; Yoshida et al., 2014) calls for exploring the psychological mechanism between servant leadership and employee creativity. Different from previous researchers who applied social learning theory (Neubert et al., 2016) and self-determination theory (Williams et al., 2017) to explain the relationship, we used COR theory with different perspectives to understand how servant leadership affects employee creativity. We determined that servant leadership has positive impacts on creativity by helping employees, including increasing their psychological safety and well-being. In turn, we used psychological safety and employee well-being as serial mediators to examine the relationship between servant leadership and creativity, contributing to the literature on servant leadership and creativity.

Second, our results validate previous literature findings such as Schaubroeck et al.’s (2011) positive relationship between servant leadership and psychological safety, but the relevant literature is limited. Our study verified the positive influence of servant leadership on psychological safety and its outcomes. For instance, Edmondson and Lei (2014) proposed that further research should be conducted on the antecedents and outcomes of psychological safety, and the results not only that servant leadership was an antecedent of psychological safety but that employee well-being and creativity were outcomes of psychological safety, which enriches the literature.

Third, we expanded the work on the relationship between servant leadership and creativity to a new context. Although many researchers have published social science works that used data conducted from participants recruited online (Bohannon, 2016), authors of the majority of extant literature in the field of servant leadership and employee performance have used traditional surveys. In contrast, for our study, we recruited participants online through an online panel platform. Previous research showed that research data obtained online are as valid, reliable, and high quality as data obtained via traditional methods (Rand, 2012; Buhrmester et al., 2016). Woods et al. (2015), and Gleibs (2017) even determined that online participants tend to be more representative of larger populations than do those recruited in traditional methods. Whereas traditional surveys are administered within specific several companies or to respondents with similar working backgrounds, which can restrict the generalizability of the results, we conducted our study online and collected data from participants from a variety of different working backgrounds. By eliminating the above restrictions, we increased the generalizability of our findings.

Finally, we extend the work on COR theory following He et al.’s (2020) application of the theory to explain the relationship between compulsory citizenship behavior and employee creativity and Braun and Peus’s (2018) use of it to explain the relationship between authentic leadership and followers’ job satisfaction. Specifically, in the relationship between servant leadership and employee creativity, we introduced the sequential mediators of psychological safety in the workplace and employee well-being: Considering psychological safety a psychological resource, we applied COR theory and used a serial mediation model to explain how servant leadership increases the sense of psychological safety, which enhances employee well-being and promotes employee creativity.

### Practical Implications

Our research has some practical implications for organizations today. First, leaders’ characteristics and behaviors greatly affect employees’ behaviors, so managers should pay more attention to employees than to their own or their organizations’ goals, and organizations should prefer leader candidates who are more interested in serving than in gaining power. These candidates are likely to become servant leaders, which can increase employees’ creativity.

Second, our research shows that the positive effect of servant leadership is transmitted through psychological safety. Usually, organizations pay more attention to the workplace atmosphere in attempting to create spaces of psychological safety, but our research indicates that leaders should be considered as well. The leader of the organization should increase the tolerance of employees’ unintentional mistakes, and give employees more opportunities to express their ideas at work, promote their career development (Jo et al., 2018) and reduce the competitive atmosphere among employees in the organization. Finally, employees often suffer from stress and burnout at work, which has negative impacts on well-being. Some businesses attempt to reduce stress and burnout and improve well-being by increasing personal income or offering career adaptability (Takao and Ishiyama, 2021), but we suggest another factor to be considered is the type of leadership (Jeong et al., 2018). We determined that the servant style of leadership has positive implications not only for employee creativity, the topic of our study, but for employees’ well-being and psychological safety as well.

### Limitations and Directions for Future Research

This study has several limitations. First, we measured variables from the same sources at two time points by self-report, following Neubert et al. (2008). Although Chan (2009) found no strong evidence that self-report prevents meaningful interpretations of study data, self-report data can show common method bias. However, Spector (2006) determined that common method bias is not the primary limiter of research result validity. We used time separation to collect the data, separating the data collection into two waves separated by 6 weeks, to reduce the risk of common method bias and used Harman’s one-factor test to examine the bias. However, using the same sources at both time points limited our ability to establish causality between variables. Future researchers should collect and study data from multiple sources for analysis.

Second, we conducted this study on an individual level to explore how employees’ perceptions of leadership affect their behavior in the workplace. However, leadership was originally a group-level construct (Hogg et al., 2005), and individual differences influence how individuals interpret and respond to their supervisors’ behaviors (Jo, 2019). Therefore, researchers still need to conduct multilevel or team-level research in the future.

Third, many scholars have investigated the stability of employee well-being over time as an issue (Warr, 1992; Kammeyer-Mueller et al., 2009; Dunford et al., 2012; Zacher et al., 2014) and found that it weakens over time; others have found that well-being has peaks and ebbs throughout an individual’s life span. Future researchers should employ a longitudinal study design to confirm the durability of employee well-being, and how the fluctuation of well-being influences employees’ behavior. Moreover, we recruited participants from the United Kingdom, and that likely also limits the generalizability of our study findings. Future researchers should expand their participant sample to different cultures such as non-Western countries.

Moreover, because pandemics can potentially affect employee well-being, which can also affect organizational performance and employee motivation, companies are increasingly focusing on their impacts on employee psychological states. Future researchers could extend this study to explore the mechanism of how servant leadership influences creativity in the context of a pandemic.

Finally, recent servant leadership studies indicate that servant leadership explains more of the outcome variables than other leadership approaches (Liden et al., 2008; Schaubroeck et al., 2011; van Dierendonck et al., 2014), but future researchers could use a different style such as transformational leadership (Eva et al., 2019) as a control to confirm how much variance in employee behavior can be explained by servant leadership.

## Data Availability Statement

The raw data supporting the conclusions of this article will be made available by the authors, without undue reservation, to any qualified researcher.

## Author Contributions

WW was the principal researcher and prepared the first draft of the article. S-WK supervised the study and refined the draft into a publishable article. In addition to motivating the publication of this article, SBC added valuable theoretical and methodological insights based on his knowledge and expertise regarding the topic. All authors have read and agreed to the submitted version of the manuscript.

## Conflict of Interest

The authors declare that the research was conducted in the absence of any commercial or financial relationships that could be construed as a potential conflict of interest.

## Publisher’s Note

All claims expressed in this article are solely those of the authors and do not necessarily represent those of their affiliated organizations, or those of the publisher, the editors and the reviewers. Any product that may be evaluated in this article, or claim that may be made by its manufacturer, is not guaranteed or endorsed by the publisher.

## Appendix A

**Servant Leadership (α = 0.84)** (Liden et al., 2015)

1. My supervisor can tell if something work-related is going wrong;
2. My supervisor makes my career development a priority;
3. I would seek help from my supervisor if I had a personal problem;
4. My supervisor emphasizes the importance of giving back to the community;
5. My supervisor puts my best interests ahead of his/her own;
6. My supervisor gives me the freedom to handle difficult situations in the way that I feel is best;
7. My supervisor would NOT compromise ethical principles in order to achieve success.

**Psychological Safety(α = 0.74)** (May et al., 2004)

1. I’m not afraid to be myself at work.
2. I’m not afraid to express my opinions at work.
3. There is no threatening environment at work.

**Employee well-being (α = 0.83)** (Brunetto et al., 2011)

1. Overall, I think being a current job worker fulfils an important purpose in my work life.
2. Overall, I get enough time in this job to reflect on what I do at work.
3. Overall I think I am reasonably satisfied with my work life.
4. Overall, most days I feel a sense of accomplishment in what I do in working.

**Creativity(α = 0.88)** (Neubert et al., 2016)

1. I generate creative ideas at work.
2. I promote and champion ideas to others.
3. I am innovative at work.

## References

[B1] Aiken L. S. West S. G. (1991). *Multiple Regression: Testing and Interpreting Interactions.* Thousand Oaks, CA: SAGE.

[B2] Amabile T. M. (1983). The social psychology of creativity: a componential conceptualization. *J. Pers. Soc. Psychol.* 45 357–376. 10.1037/0022-3514.45.2.357

[B3] Amabile T. M. Barsade S. G. Mueller J. S. Staw B. M. (2005). Affect and creativity at work. *Adm. Sci. Q.* 50 367–403. 10.2189/asqu.2005.50.3.367 21821037

[B4] Axtell C. M. Holman D. J. Unsworth K. L. Wall T. D. Waterson P. E. Harrington E. (2000). Shopfloor innovation: facilitating the suggestion and implementation of ideas. *J. Occup. Organ. Psychol.* 73 265–285. 10.1348/096317900167029

[B5] Bakker A. Oerlemans W. (2012). *Subjective Well-Being in Organizations.* New York, NY: Oxford University.

[B6] Black G. L. (2010). Correlational analysis of servant leadership and school climate. *Cathol. Educ. J. Inq. Pract.* 13:3.

[B7] Bohannon J. (2016). Mechanical Turk upends social sciences. *Science* 352 1263–1264. 27284175 10.1126/science.352.6291.1263

[B8] Braun S. Peus C. (2018). Crossover of work–life balance perceptions: does authentic leadership matter? *J. Bus. Ethics* 149 875–893. 10.1007/s10551-016-3078-x

[B9] Brown S. P. Leigh T. W. (1996). A new look at psychological climate and its relationship to job involvement, effort, and performance. *J. Appl. Psychol.* 81 358–368. 10.1037/0021-9010.81.4.358 8751453

[B10] Brunetto Y. Farr-Wharton R. Shacklock K. (2011). Supervisor-nurse relationships, teamwork, role ambiguity and well-being: public versus private sector nurses. *Asia Pac. J. Hum. Resour.* 49 143–164. 10.1177/1038411111400161

[B11] Buhrmester M. Kwang T. Gosling S. D. (2016). *Amazon’s Mechanical Turk: A New Source of Inexpensive, Yet High-Quality Data?.* Washington, DC: American Psychological Association. 10.1177/1745691610393980 26162106

[B12] Carlson M. Charlin V. Miller N. (1988). Positive mood and helping behavior: a test of six hypotheses. *J. Pers. Soc. Psychol.* 55 211–229. 10.1037/0022-3514.55.2.211 3050025

[B13] Carmeli A. Sheaffer Z. Binyamin G. Reiter-Palmon R. Shimoni T. (2014). Transformational leadership and creative problem-solving: the mediating role of psychological safety and reflexivity. *J. Creat. Behav.* 48 115–135. 10.1002/jocb.43

[B14] Chan D. (2009). “So why ask me? Are self-report data really that bad?,” in *Statistical and Methodological Myths and Urban Legends: Doctrine, Verity and Fable in the Organizational and Social Sciences*, eds Lance C. E. Vandenberg R. J. (New York, NY: Routledge/Taylor & Francis Group), 309–336.

[B15] Chang S.-J. van Witteloostuijn A. Eden L. (2010). From the editors: common method variance in international business research. *J. Int. Bus. Stud.* 41 178–184. 10.1057/jibs.2009.88

[B16] Chen C. Liao J. Wen P. (2014). Why does formal mentoring matter? The mediating role of psychological safety and the moderating role of power distance orientation in the Chinese context. *Int. J. Hum. Resour. Manag.* 25 1112–1130. 10.1080/09585192.2013.816861

[B17] Chen C.-Y. Chen C.-H. V. Li C.-I. (2013). The influence of leader’s spiritual values of servant leadership on employee motivational autonomy and eudaemonic well-being. *J. Relig. Health* 52 418–438. 10.1007/s10943-011-9479-3 21424861

[B18] Cheung J. H. Burns D. K. Sinclair R. R. Sliter M. (2017). Amazon mechanical turk in organizational psychology: an evaluation and practical recommendations. *J. Bus. Psychol.* 32 347–361. 10.1007/s10869-016-9458-5

[B19] Chughtai A. A. (2016). Servant leadership and follower outcomes: mediating effects of organizational identification and psychological safety. *J. Psychol.* 150 866–880. 10.1080/00223980.2016.1170657 27101125

[B20] Dirik H. F. Intepeler S. S. (2017). The influence of authentic leadership on safety climate in nursing. *J. Nurs. Manag.* 25 392–401. 10.1111/jonm.12480 28543942

[B21] Dunford B. B. Shipp A. J. Boss R. W. Angermeier I. Boss A. D. (2012). Is burnout static or dynamic? A career transition perspective of employee burnout trajectories. *J. Appl. Psychol.* 97 637–650. 10.1037/a0027060 22309410

[B22] Edmondson A. (1999). Psychological safety and learning behavior in work teams. *Adm. Sci. Q.* 44 350–383. 10.2307/2666999

[B23] Edmondson A. C. (2004). “Psychological safety, trust, and learning in organizations: a group-level lens,” in *Trust and Distrust in Organizations: Dilemmas and Approaches The Russell Sage Foundation Series on Trust*, eds Kramer R. M. Cook K. S. (New York, NY: Russell Sage Foundation), 239–272.

[B24] Edmondson A. C. (2018). *The Fearless Organization: Creating Psychological Safety in the Workplace for Learning, Innovation, and Growth.* Hoboken, NJ: John Wiley & Sons.

[B25] Edmondson A. C. Lei Z. (2014). Psychological safety: the history, renaissance, and future of an interpersonal construct. *Annu. Rev. Organ. Psychol. Organ. Behav.* 1 23–43. 10.1146/annurev-orgpsych-031413-091305

[B26] Elche D. Ruiz-Palomino P. Linuesa-Langreo J. (2020). Servant leadership and organizational citizenship behavior: the mediating effect of empathy and service climate. *Int. J. Contemp. Hosp. Manag.* 32 2035–2053. 10.1108/IJCHM-05-2019-0501

[B27] Erkutlu H. Chafra J. (2019). Leader psychopathy and organizational deviance: the mediating role of psychological safety and the moderating role of moral disengagement. *Int. J. Workplace Health Manag.* 12 197–213. 10.1108/IJWHM-12-2018-0154

[B28] Eva N. Robin M. Sendjaya S. van Dierendonck D. Liden R. C. (2019). Servant leadership: a systematic review and call for future research. *Leadersh. Q.* 30 111–132. 10.1016/j.leaqua.2018.07.004

[B29] Frazier M. L. Fainshmidt S. Klinger R. L. Pezeshkan A. Vracheva V. (2017). Psychological safety: a meta-analytic review and extension. *Pers. Psychol.* 70 113–165. 10.1111/peps.12183

[B30] Fuller C. M. Simmering M. J. Atinc G. Atinc Y. Babin B. J. (2016). Common methods variance detection in business research. *J. Bus. Res.* 69 3192–3198. 10.1016/j.jbusres.2015.12.008

[B31] George J. M. (1991). State or trait: effects of positive mood on prosocial behaviors at work. *J. Appl. Psychol.* 76 299–307. 10.1037/0021-9010.76.2.299

[B32] George J. M. (2007). Creativity in organizations. *Acad. Manag. Ann.* 1 439–477. 10.1080/078559814

[B33] Gleibs I. H. (2017). Are all “research fields” equal? Rethinking practice for the use of data from crowdsourcing market places. *Behav. Res. Methods* 49 1333–1342. 10.3758/s13428-016-0789-y 27515317 PMC5541108

[B34] Gong Y. Kim T.-Y. Lee D.-R. Zhu J. (2013). A multilevel model of team goal orientation, information exchange, and creativity. *Acad. Manage. J.* 56 827–851. 10.5465/amj.2011.0177

[B35] Gotsis G. Grimani K. (2016). The role of servant leadership in fostering inclusive organizations. *J. Manag. Dev.* 35 985–1010. 10.1108/JMD-07-2015-0095

[B36] Grant A. M. Green S. Rynsaardt J. (2010). Developmental coaching for high school teachers: executive coaching goes to school. *Consult. Psychol. J. Pract. Res.* 62 151–168. 10.1037/a0019212

[B37] Greenleaf R. (2007). “The Servant as Leader,” in *Corporate Ethics and Corporate Governance*, eds Zimmerli W. C. Holzinger M. Richter K. (Berlin: Springer), 79–85.

[B38] Greenleaf R. K. (1977). *Servant Leadership: A Journey into the Nature of Legitimate Power and Greatness.* New York, NY: Paulist Press.

[B39] Halbesleben J. R. B. Neveu J.-P. Paustian-Underdahl S. C. Westman M. (2014). Getting to the “COR”: understanding the role of resources in conservation of resources theory. *J. Manag.* 40 1334–1364. 10.1177/0149206314527130

[B40] Hasan F. Kashif M. (2020). Psychological safety, meaningfulness and empowerment as predictors of employee well-being: a mediating role of promotive voice. *Asia Pac. J. Bus. Adm.* 13 40–59. 10.1108/APJBA-11-2019-0236

[B41] He P. Zhou Q. Zhao H. Jiang C. Wu Y. J. (2020). Compulsory citizenship behavior and employee creativity: creative self-efficacy as a mediator and negative affect as a moderator. *Front. Psychol.* 11:1640. 10.3389/fpsyg.2020.01640 32793046 PMC7385136

[B42] Hobfoll S. E. (1989). Conservation of resources. A new attempt at conceptualizing stress. *Am. Psychol.* 44 513–524. 10.1037//0003-066x.44.3.513 2648906

[B43] Hobfoll S. E. Halbesleben J. Neveu J.-P. Westman M. (2018). Conservation of resources in the organizational context: the reality of resources and their consequences. *Annu. Rev. Organ. Psychol. Organ. Behav.* 5 103–128. 10.1146/annurev-orgpsych-032117-104640

[B44] Hoch J. E. Bommer W. H. Dulebohn J. H. Wu D. (2018). Do ethical, authentic, and servant leadership explain variance above and beyond transformational leadership? A meta-analysis. *J. Manag.* 44 501–529. 10.1177/0149206316665461

[B45] Hogg M. A. Martin R. Epitropaki O. Mankad A. Svensson A. Weeden K. (2005). Effective leadership in salient groups: revisiting leader-member exchange theory from the perspective of the social identity theory of leadership. *Pers. Soc. Psychol. Bull.* 31 991–1004. 10.1177/0146167204273098 15951369

[B46] Howell J. M. Neufeld D. J. Avolio B. J. (2005). Examining the relationship of leadership and physical distance with business unit performance. *Leadersh. Q.* 16 273–285. 10.1016/j.leaqua.2005.01.004

[B47] Hughes D. J. Lee A. Tian A. W. Newman A. Legood A. (2018). Leadership, creativity, and innovation: a critical review and practical recommendations. *Leadersh. Q.* 29 549–569. 10.1016/j.leaqua.2018.03.001

[B48] Iqbal A. Latif K. F. Ahmad M. S. (2020). Servant leadership and employee innovative behaviour: exploring psychological pathways. *Leadersh. Organ. Dev. J.* 41 813–827. 10.1108/LODJ-11-2019-0474

[B49] Janssen O. (2000). Job demands, perceptions of effort-reward fairness and innovative work behaviour. *J. Occup. Organ. Psychol.* 73 287–302. 10.1348/096317900167038

[B50] Jaramillo F. Grisaffe D. B. Chonko L. B. Roberts J. A. (2009). Examining the Impact of Servant Leadership on Sales Force Performance. *J. Pers. Sell. Sales Manag.* 29 257–275. 10.2753/PSS0885-3134290304

[B51] Jeong S. E. Choi B. Chung T.-Y. (2018). *The Foundation of Business Administration.* Korea: Harin Book Publishing Uijeongbu.

[B52] Jo S. J. (2019). *History of Business and Management.* Seoul: Hankyung Book Publishing.

[B53] Jo S. J. Bae E. G. Kim H. S. Kim D. Y. Lee M. Y. Rhee S. S. (2018). *Models for HRD Practice: Career Development.* Seoul: Parkyoungsa Book Publishing.

[B54] Kahn W. A. (1990). Psychological conditions of personal engagement and disengagement at work. *Acad. Manage. J.* 33 692–724. 10.5465/256287

[B55] Kammeyer-Mueller J. D. Judge T. A. Scott B. A. (2009). The role of core self-evaluations in the coping process. *J. Appl. Psychol.* 94 177–195. 10.1037/a0013214 19186903

[B56] Karatepe O. M. Ozturk A. Kim T. T. (2019). Servant leadership, organisational trust, and bank employee outcomes. *Serv. Ind. J.* 39 86–108. 10.1080/02642069.2018.1464559

[B57] Khan M. M. Mubarik M. S. Islam T. (2020). Leading the innovation: role of trust and job crafting as sequential mediators relating servant leadership and innovative work behavior. *Eur. J. Innov. Manag.* [Epub ahead of print]. 10.1108/EJIM-05-2020-0187

[B58] Kirk-Brown A. Van Dijk P. (2016). An examination of the role of psychological safety in the relationship between job resources, affective commitment and turnover intentions of Australian employees with chronic illness. *Int. J. Hum. Resour. Manag.* 27 1626–1641. 10.1080/09585192.2015.1053964

[B59] Lee H. W. Choi J. N. Kim S. (2018). Does gender diversity help teams constructively manage status conflict? An evolutionary perspective of status conflict, team psychological safety, and team creativity. *Organ. Behav. Hum. Decis. Process.* 144 187–199. 10.1016/j.obhdp.2017.09.005

[B60] Liden R. C. Wayne S. J. Liao C. Meuser J. D. (2014). Servant leadership and serving culture: influence on individual and unit performance. *Acad. Manage. J.* 57 1434–1452. 10.5465/amj.2013.0034

[B61] Liden R. C. Wayne S. J. Meuser J. D. Hu J. Wu J. Liao C. (2015). Servant leadership: validation of a short form of the SL-28. *Leadersh. Q.* 26 254–269. 10.1016/j.leaqua.2014.12.002

[B62] Liden R. C. Wayne S. J. Zhao H. Henderson D. (2008). Servant leadership: development of a multidimensional measure and multi-level assessment. *Leadersh. Q.* 19 161–177. 10.1016/j.leaqua.2008.01.006

[B63] Linuesa-Langreo J. Ruiz-Palomino P. Elche D. (2016). Servant leadership, empowerment climate, and group creativity_ a case study in the hospitality industry. *Ramon Llull J. Appl. Ethics* 7 9–36.

[B64] Liu D. Liao H. Loi R. (2012). The dark side of leadership: a three-level investigation of the cascading effect of abusive supervision on employee creativity. *Acad. Manage. J.* 55 1187–1212. 10.5465/amj.2010.0400

[B65] Liu K. Ge Y. (2020). How psychological safety influences employee creativity in China: work engagement as a mediator. *Soc. Behav. Personal.* 48:e9211. 10.2224/sbp.9211

[B66] Liu W. Zhang P. Liao J. Hao P. Mao J. (2016). Abusive supervision and employee creativity: the mediating role of psychological safety and organizational identification. *Manag. Decis.* 54 130–147. 10.1108/MD-09-2013-0443

[B67] Lyu X. (2016). Effect of organizational justice on work engagement with psychological safety as a mediator: evidence from China. *Soc. Behav. Personal. Int. J.* 44 1359–1370. 10.2224/sbp.2016.44.8.1359

[B68] Mai K. M. Welsh D. T. Wang F. Bush J. Jiang K. (2021). Supporting creativity or creative unethicality? empowering leadership and the role of performance pressure. *J. Bus. Ethics* [Epub ahead of print]. 10.1007/s10551-021-04784-6

[B69] Marjanovic Z. Struthers C. W. Cribbie R. Greenglass E. R. (2014). The conscientious responders scale: a new tool for discriminating between conscientious and random responders. *SAGE Open* 4:2158244014545964. 10.1177/2158244014545964

[B70] Marsh H. W. Hau K.-T. Wen Z. (2004). In search of golden rules: comment on hypothesis-testing approaches to setting cutoff values for fit indexes and dangers in overgeneralizing Hu and Bentler’s (1999) findings. *Struct. Equ. Model. Multidiscip. J.* 11 320–341. 10.1207/s15328007sem1103_2

[B71] May D. R. Gilson R. L. Harter L. M. (2004). The psychological conditions of meaningfulness, safety and availability and the engagement of the human spirit at work. *J. Occup. Organ. Psychol.* 77 11–37. 10.1348/096317904322915892

[B72] Mayer D. M. Bardes M. Piccolo R. F. (2008). Do servant-leaders help satisfy follower needs? An organizational justice perspective. *Eur. J. Work Organ. Psychol.* 17 180–197. 10.1080/13594320701743558

[B73] Miao R. Cao Y. (2019). High-performance work system, work well-being, and employee creativity: cross-level moderating role of transformational leadership. *Int. J. Environ. Res. Public. Health* 16:1640. 10.3390/ijerph16091640 31083469 PMC6539597

[B74] Moneta G. B. Amabile T. M. Schatzel E. A. Kramer S. J. (2010). Multirater assessment of creative contributions to team projects in organizations. *Eur. J. Work Organ. Psychol.* 19 150–176. 10.1080/13594320902815312

[B75] Neubert M. J. Hunter E. M. Tolentino R. C. (2016). A servant leader and their stakeholders: when does organizational structure enhance a leader’s influence? *Leadersh. Q.* 27 896–910. 10.1016/j.leaqua.2016.05.005

[B76] Neubert M. J. Kacmar K. M. Carlson D. S. Chonko L. B. Roberts J. A. (2008). Regulatory focus as a mediator of the influence of initiating structure and servant leadership on employee behavior. *J. Appl. Psychol.* 93 1220–1233. 10.1037/a0012695 19025244

[B77] Newman A. Donohue R. Eva N. (2017). Psychological safety: a systematic review of the literature. *Hum. Resour. Manag. Rev.* 27 521–535. 10.1016/j.hrmr.2017.01.001

[B78] Newman A. Neesham C. Manville G. Tse H. H. M. (2018). Examining the influence of servant and entrepreneurial leadership on the work outcomes of employees in social enterprises. *Int. J. Hum. Resour. Manag.* 29 2905–2926. 10.1080/09585192.2017.1359792

[B79] Oldham G. R. Cummings A. (1996). Employee creativity: personal and contextual factors at work. *Acad. Manage. J.* 39 607–634. 10.2307/256657

[B80] Oppenheimer D. M. Meyvis T. Davidenko N. (2009). Instructional manipulation checks: detecting satisficing to increase statistical power. *J. Exp. Soc. Psychol.* 45 867–872. 10.1016/j.jesp.2009.03.009

[B81] Palan S. Schitter C. (2018). Prolific.ac—A subject pool for online experiments. *J. Behav. Exp. Finance* 17 22–27. 10.1016/j.jbef.2017.12.004

[B82] Peer E. Brandimarte L. Samat S. Acquisti A. (2017). Beyond the turk: alternative platforms for crowdsourcing behavioral research. *J. Exp. Soc. Psychol.* 70 153–163. 10.1016/j.jesp.2017.01.006

[B83] Peer E. Vosgerau J. Acquisti A. (2014). Reputation as a sufficient condition for data quality on Amazon Mechanical Turk. *Behav. Res. Methods* 46 1023–1031. 10.3758/s13428-013-0434-y 24356996

[B84] Podsakoff P. M. MacKenzie S. B. Lee J.-Y. Podsakoff N. P. (2003). Common method biases in behavioral research: a critical review of the literature and recommended remedies. *J. Appl. Psychol.* 88 879–903. 10.1037/0021-9010.88.5.879 14516251

[B85] Preacher K. J. Hayes A. F. (2008). Asymptotic and resampling strategies for assessing and comparing indirect effects in multiple mediator models. *Behav. Res. Methods* 40 879–891. 10.3758/BRM.40.3.879 18697684

[B86] Rand D. G. (2012). The promise of mechanical turk: how online labor markets can help theorists run behavioral experiments. *J. Theor. Biol.* 299 172–179. 10.1016/j.jtbi.2011.03.004 21402081

[B87] Ruiz-Palomino P. Yáñez-Araque B. Jiménez-Estévez P. Gutiérrez-Broncano S. (2021). Can servant leadership prevent hotel employee depression during the COVID-19 pandemic? A mediating and multigroup analysis. *Technol. Forecast. Soc. Change* 174:121192. 10.1016/j.techfore.2021.121192 34538969 PMC8437813

[B88] Ruiz-Palomino P. Zoghbi-Manrique-de-Lara P. (2020). How and when servant leaders fuel creativity: the role of servant attitude and intrinsic motivation. *Int. J. Hosp. Manag.* 89:102537. 10.1016/j.ijhm.2020.102537

[B89] Russell R. F. (2001). The role of values in servant leadership. *Leadersh. Organ. Dev. J.* 22 76–84. 10.1108/01437730110382631

[B90] Ryan R. M. Deci E. L. (2001). On happiness and human potentials: a review of research on hedonic and eudaimonic well-being. *Annu. Rev. Psychol.* 52 141–166. 10.1146/annurev.psych.52.1.141 11148302

[B91] Schaubroeck J. Lam S. S. K. Peng A. C. (2011). Cognition-based and affect-based trust as mediators of leader behavior influences on team performance. *J. Appl. Psychol.* 96 863–871. 10.1037/a0022625 21299271

[B92] Schepers J. de Jong A. Wetzels M. de Ruyter K. (2008). Psychological safety and social support in groupware adoption: a multi-level assessment in education. *Comput. Educ.* 51 757–775. 10.1016/j.compedu.2007.08.001

[B93] Schneider S. K. George W. M. (2011). Servant leadership versus transformational leadership in voluntary service organizations. *Leadersh. Organ. Dev. J.* 32 60–77. 10.1108/01437731111099283

[B94] Shalley C. E. Gilson L. L. (2004). What leaders need to know: a review of social and contextual factors that can foster or hinder creativity. *Leadersh. Q.* 15 33–53. 10.1016/j.leaqua.2003.12.004

[B95] Shalley C. E. Gilson L. L. Blum T. C. (2009). Interactive effects of growth need strength, work context, and job complexity on self-reported creative performance. *Acad. Manage. J.* 52 489–505. 10.5465/amj.2009.41330806

[B96] Shalley C. E. Zhou J. Oldham G. R. (2004). The effects of personal and contextual characteristics on creativity: where should we go from here? *J. Manag.* 30 933–958. 10.1016/j.jm.2004.06.007

[B97] Sharifirad M. S. (2013). Transformational leadership, innovative work behavior, and employee well-being. *Glob. Bus. Perspect.* 3 198–225. 10.1007/s40196-013-0019-2

[B98] Singh B. Shaffer M. A. Selvarajan T. T. (2018). Antecedents of organizational and community embeddedness: the roles of support, psychological safety, and need to belong. *J. Organ. Behav.* 39 339–354. 10.1002/job.2223

[B99] Sonnentag S. (2015). Dynamics of well-being. *Annu. Rev. Organ. Psychol. Organ. Behav.* 2 261–293. 10.1146/annurev-orgpsych-032414-111347

[B100] Spector P. E. (2006). Method variance in organizational research: truth or urban legend? *Organ. Res. Methods* 9 221–232. 10.1177/1094428105284955

[B101] Spreitzer G. Porath C. L. Gibson C. B. (2012). Toward human sustainability: how to enable more thriving at work. *Organ. Dyn.* 41 155–162. 10.1016/j.orgdyn.2012.01.009

[B102] Sprigg C. A. Niven K. Dawson J. Farley S. Armitage C. J. (2018). Witnessing workplace bullying and employee well-being: a two-wave field study. *J. Occup. Health Psychol.* 24 286–296. 10.1037/ocp0000137 30489100

[B103] Takao M. Ishiyama N. (2021). Effect of career adaptability on subjective well-being of middle-aged and older employees. *Sustainability* 13:2570. 10.3390/su13052570

[B104] Tierney P. Farmer S. M. (2011). Creative self-efficacy development and creative performance over time. *J. Appl. Psychol.* 96 277–293. 10.1037/a0020952 20954756

[B105] Tong E. M. W. Lum D. J. K. Sasaki E. Yu Z. (2019). Concurrent and temporal relationships between humility and emotional and psychological well-being. *J. Happiness Stud.* 20 1343–1358. 10.1007/s10902-018-0002-3

[B106] van Dierendonck D. (2011). Servant leadership: a review and synthesis. *J. Manag.* 37 1228–1261. 10.1177/0149206310380462

[B107] van Dierendonck D. Stam D. Boersma P. de Windt N. Alkema J. (2014). Same difference? Exploring the differential mechanisms linking servant leadership and transformational leadership to follower outcomes. *Leadersh. Q.* 25 544–562. 10.1016/j.leaqua.2013.11.014

[B108] Walter S. L. Seibert S. E. Goering D. O’Boyle E. H. (2019). A tale of two sample sources: do results from online panel data and conventional data converge? *J. Bus. Psychol.* 34 425–452. 10.1007/s10869-018-9552-y

[B109] Wang Y. Liu J. Zhu Y. (2018). Humble leadership, psychological safety, knowledge sharing, and follower creativity: a cross-level investigation. *Front. Psychol.* 9:1727. 10.3389/fpsyg.2018.01727 30283379 PMC6156152

[B110] Warr P. (1987). *Work, Unemployment, and Mental Health.* New York, NY: Oxford University Press.

[B111] Warr P. (1992). Age and occupational well-being. *Psychol. Aging* 7 37–45. 10.1037//0882-7974.7.1.37 1558703

[B112] Williams W. A. Brandon R.-S. Hayek M. Haden S. P. Atinc G. (2017). Servant leadership and followership creativity: the influence of workplace spirituality and political skill. *Leadersh. Organ. Dev. J.* 38 178–193. 10.1108/LODJ-02-2015-0019

[B113] Woods A. T. Velasco C. Levitan C. A. Wan X. Spence C. (2015). Conducting perception research over the internet: a tutorial review. *PeerJ* 3:e1058. 10.7717/peerj.1058 26244107 PMC4517966

[B114] Xu J. Xie B. Tang B. (2020). Guanxi HRM practice and employees’ occupational well-being in china: a multi-level psychological process. *Int. J. Environ. Res. Public. Health* 17:2403. 10.3390/ijerph17072403 32244746 PMC7178135

[B115] Yam K. C. Fehr R. Keng-Highberger F. T. Klotz A. C. Reynolds S. J. (2016). Out of control: a self-control perspective on the link between surface acting and abusive supervision. *J. Appl. Psychol.* 101 292–301. 10.1037/apl0000043 26214087

[B116] Yang J. Gu J. Liu H. (2019). Servant leadership and employee creativity: the roles of psychological empowerment and work–family conflict. *Curr. Psychol.* 38 1417–1427. 10.1007/s12144-019-0161-3

[B117] Yang J. Liu H. Gu J. (2017). A multi-level study of servant leadership on creativity: the roles of self-efficacy and power distance. *Leadersh. Organ. Dev. J.* 38 610–629. 10.1108/LODJ-10-2015-0229

[B118] Yoshida D. T. Sendjaya S. Hirst G. Cooper B. (2014). Does servant leadership foster creativity and innovation? A multi-level mediation study of identification and prototypicality. *J. Bus. Res.* 67 1395–1404. 10.1016/j.jbusres.2013.08.013

[B119] Zacher H. Jimmieson N. L. Bordia P. (2014). Time pressure and coworker support mediate the curvilinear relationship between age and occupational well-being. *J. Occup. Health Psychol.* 19 462–475. 10.1037/a0036995 24885685

[B120] Zhang Z. Song P. (2020). Multi-level effects of humble leadership on employees’ work well-being: the roles of psychological safety and error management climate. *Front. Psychol.* 11, 3150. 10.3389/fpsyg.2020.571840 33262726 PMC7685992

[B121] Zhu J. Xu S. Zhang B. (2020). The paradoxical effect of inclusive leadership on subordinates’ creativity. *Front. Psychol.* 10:2960. 10.3389/fpsyg.2019.02960 32038369 PMC6988563

[B122] Zoghbi-Manrique-de-Lara P. Ruiz-Palomino P. (2019). How servant leadership creates and accumulates social capital personally owned in hotel firms. *Int. J. Contemp. Hosp. Manag.* 31 3192–3211. 10.1108/IJCHM-09-2018-0748

